# Postβ-Lactamase-Inhibiting Effect of Sulbactam in Combination with Ceftriaxone on Extended-Spectrum-β-Lactamase-Producing *Escherichia coli*

**DOI:** 10.3390/antibiotics14090915

**Published:** 2025-09-11

**Authors:** Ru Wang, Kun Mi, Aihua Lu, Chengyang Zhang, Lei Sun, Yuxiang Chen, Yuanhu Pan, Yanfei Tao, Lingli Huang

**Affiliations:** 1 MOA Laboratory of Risk Assessment for Quality and Safety of Livestock and Poultry Products, Wuhan 430070, China; w1665790632@webmail.hzau.edu.cn (R.W.); mi.kun@ufl.edu (K.M.); xxzz@webmail.hzau.edu.cn (C.Z.); sunlei23@webmail.hazu.edu.cn (L.S.); cyx0426@webmail.hzau.edu.cn (Y.C.); panyuanhu@mail.hzau.edu.cn (Y.P.); tyf@mail.hzau.edu.cn (Y.T.); 2 National Reference Laboratory of Veterinary Drug Residues (HZAU) and MAO Key Laboratory for the Detection of Veterinary Drug Residues, Wuhan 430070, China; 3 Guangdong Hengqin Xinchuangyi Biomedical Co., Ltd., Zhuhai 519000, China; lhai0609@126.com; 4 Hubei Collaborative Innovation Center for Animal Nutrition and Feed Safety, Huazhong Agricultural University, Wuhan 430070, China

**Keywords:** ceftriaxone, sulbactam, postβ-lactamase inhibitor effect, hollow-fiber infection model

## Abstract

**Background/Objectives**: Extended-spectrum β-lactamase (ESBL)-producing *Escherichia coli* poses a significant global health challenge, as it leads to antimicrobial treatment failure and is associated with elevated mortality rates. The use of β-lactam/β-lactamase inhibitor combinations offers an alternative approach for combating ESBL-producing bacteria. Ceftriaxone (CRO) and sulbactam have been coadministered in the clinical settings; however, discrepancies in their pharmacokinetics raise concerns regarding the rationality of this combination. **Methods**: This study was designed to investigate the postβ-lactamase inhibitor effect (PLIE) under both static and dynamic conditions, with the aim of supporting the clinical application of this combination. **Results**: The minimum inhibitory concentration (MIC) of CRO/SBT (2:1 ratio) against *E. coli* NCTC 13353 was determined to be 32/16 μg/mL. The PLIEs were determined to be −1.26, −0.57, and 0.37 h at CRO/SBT concentrations ranging from 1/2 MIC to 2 MIC, respectively. The results of CRO concentration, β-lactamase activity, *bla_CTX-M-15_* expression, and cell morphology collectively support that SBT exerts PLIEs and protects against the antibacterial activity of CRO. In the dynamic hollow-fiber infection model, CRO monotherapy showed no inhibitory effect on *E. coli*, whereas CRO/SBT combination therapy rapidly eliminated SBT, achieved comparable bactericidal effects, prolonged CRO exposure, and maintained low β-lactamase activity levels. **Conclusions**: In conclusion, CRO/SBT exerts an inhibitory effect on enzyme-producing strains by being able to produce PLIE to maintain the inhibition of β-lactamase.

## 1. Introduction

Ceftriaxone (CRO), a third-generation cephalosporin, demonstrates potent activity against Gram-negative bacteria, including *Haemophilus influenzae* and *Escherichia coli*, as well as most Gram-positive bacteria such as *Streptococcus pneumoniae* [1,2,3]. It is frequently used to treat respiratory, urinary tract, soft tissue, and joint infections; bacterial meningitis; and gonorrhea [4,5]. Approximately 45–60% of CRO is excreted in urine, and the remainder is excreted via the biliary pathway [6]. It has a prolonged half-life (7.60–9.05 h), which supports once-daily dosing via intravenous or intramuscular administration [7]. However, in recent years, the rising incidence of infections caused by third-generation cephalosporin-resistant enterobacteria has posed a significant risk to public health [8].

Extended-spectrum β-lactamases (ESBLs), which are typically carried by mobile genetic elements, are key mechanisms responsible for resistance to cephalosporins, facilitating their dissemination and producing hydrolytic activity, thus increasing resistance [9,10,11]. ESBLs include the TEM, SHV, CTX-M, and OXA types. Among them, the CTX-M type, particularly CTX-M-14 and CTX-M-15, belongs to class A β-lactamase and represents the most prevalent genotype of ESBLs in China. Patli et al. reported that all ESBL-producing *E. coli* isolates (n = 50) were completely resistant to ceftriaxone (CRO), with the predominant genotype being a common CTX-M subtype [12]. The combination of β-lactam and β-lactamase inhibitors (BLIs) is increasingly considered an alternative strategy to treat ESBL-producing infections.

Sulbactam (SBT), an irreversible inhibitor of β-lactamase, prevents the binding of β-lactamases to β-lactams, especially classes A and C [13], thereby extending the antimicrobial activity of β-lactams to include resistant bacterial strains [14]. Compared with CRO monotherapy, the CRO-SBT combination at a 2:1 ratio has been reported to have a significant effect on ultrabroad-spectrum β-lactamase-producing bacteria, such as *Escherichia coli* [15,16,17,18]. SBT has been commercially available in combination with CRO (EXTACEF^®^-XL). However, this combination raises doubts because of the much shorter half-life of SBT, approximately 1 h [19], than that of CRO, resulting in a pharmacokinetic (PK) mismatch and leaving CRO unprotected against β-lactamase. Nevertheless, the observed clinical efficacy of CRO/SBT combinations suggests that additional mechanisms may contribute to their antibacterial activity [20].

The postβ-lactamase inhibitor effect (PLIE) describes the continued inhibition of β-lactam antibiotics on β-lactamase-producing bacteria after β-lactamase elimination [21]. PLIE is particularly significant in the context of β-lactam/β-lactamase inhibitor combinations, as it improves the pharmacodynamic interpretation and offers a theoretical rationale for the observed synergistic effects, particularly when the pharmacokinetics of the two components are mismatched [22]. To date, the PLIE is reflected only by the determination of bacterial growth after removing β-lactamase inhibitors at a static concentration, which means that CRO or SBT maintains a fixed concentration during the experiment [21,22,23,24]. However, critical factors such as drug concentrations, β-lactamase activity, and gene expression levels are ignored. Furthermore, the changes in these factors under dynamic concentrations remains unexplored.

In the present study, the PLIEs of CRO/SBT combinations were evaluated under static and dynamic conditions. First, under static conditions, we assessed bacterial growth, drug concentrations, β-lactamase activities, gene expression levels, and bacterial morphology following treatment with CRO monotherapy and CRO/SBT combination therapy at various ratios and determined the corresponding PLIE. Second, hollow fiber infection (HFIM) models were established to simulate the in vivo PK of CRO monotherapy and CRO/SBT combinations. Finally, using these dynamic models, we determined the time-course changes in drug concentrations, bacterial burden, and β-lactamase activity under different dosing ratios to evaluate the PLIE. This study provides a comprehensive evaluation of PLIE and novel insight into the synergistic effects of CRO/SBT combinations, supporting its application.

## 2. Results

### 2.1. Susceptibility of E. coli NCTC 13353 to CRO/SBT

The MICs of CRO, CRO/SBT (2:1), and CRO/SBT (fixed SBT concentration of 4 µg/mL) against ultrabroad-spectrum β-lactamase-producing *E. coli* NCTC 13353 are >1024 µg/mL, 32/16 µg/mL, and 128/4 µg/mL, respectively. SBT increased the antimicrobial effect of CRO at a ratio of 2:1, and the MIC decreased 32-fold.

### 2.2. SBT Can Induce PLIE and Improve the Antibacterial Effect of CRO Under Static Conditions

**Figure 1:**
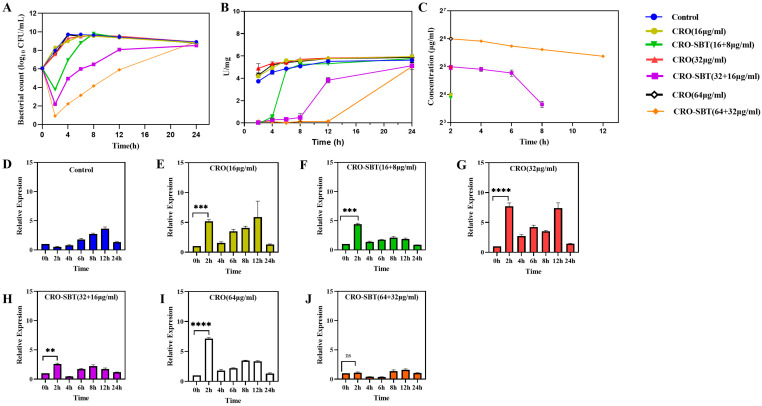
Determination of bacterial count (Panel (**A**)), beta-lactamase activity (Panel (**B**)), ceftriaxone concentration (Panel (**C**)), and relative *bla_CTX-M-15_* gene expression levels using the initial time point as the reference for the Control group (Panel (**D**)), CRO 16 µg/mL (Panel (**E**)), CRO/SBT 16/8 µg/mL (Panel (**F**)), CRO 32 µg/mL (Panel (**G**)), CRO/SBT 32/16 µg/mL (Panel (**H**)), CRO 64 µg/mL (Panel (**I**)), and CRO/SBT 64/32 µg/mL (Panel (**J**)). ** *p* < 0.01, *** *p* < 0.001, **** *p* < 0.0001 vs. control group.

After removing CRO and CRO/SBT and only adding the same concentration of CRO, the bacterial kinetic curves were determined, as shown in Figure 1A. The SBT concentrations were determined by the developed HPLC-MS/MS which is under the limit of detection indicating the effective remove. The growth trends of *E. coli* in the CRO monotherapy group and the control group were comparable. CRO alone lacked antimicrobial activity on *E. coli* NCTC 13353. In contrast, after exposure to CRO/SBT for 2 h, the bacterial counts were reduced by 2.3, 3.9, and 5.1 log_10_ CFU/mL in the combination groups at 1/2MIC, 1MIC, and 2MIC, respectively. Additionally, the time required for the regrowth of *E. coli* to the initial inoculum level (10^6^ CFU/mL) was 4 h, 8 h, and 12 h in the combination group at CRO concentrations of 1/2MIC, 1MIC, and 2MIC, respectively. SBT was found to induce PLIE, which was quantified as −1.26, −0.57, and 0.37 h for CRO/SBT treatments of 1/2MIC, 1MIC, and 2MIC, respectively. There is a concentration-dependent PLIE, as the PLIE increases with increasing SBT concentration.

β-lactamase activities were also determined during PLIE (Figure 1B). In the CRO monotherapy group, the β-lactamase activities were similar to those in the control group. In the combination group, after removing SBT, the β-lactamase activities were initially inhibited because of the PLIE of SBT. The β-lactamase activities were gradually recovered to the level of the control group. The degradation rate of β-lactamase to CRO also reflected β-lactamase activity (Figure 1C). CRO was completely degraded within 2 h in both the single CRO treatment group and the CRO/SBT (16:8) combination treatment group. In contrast, the degradation of CRO was slower in the CRO/SBT combination groups at 32/16 µg/mL and 64/32 µg/mL when β-lactamase activity was limited, whereas the degradation rate increased as β-lactamase activity gradually recovered. CRO concentration in 32/16 µg/mL group remained at 12.58 ± 0.98 µg/mL at the 8-h time point but became undetectable at 12 h. In the group of 64/32 µg/mL, the CRO concentration was 41.53 ± 0.70 µg/mL at the 12 h and completely degraded before 24 h.

The relative expression levels of the *bla_CTX-M-15_* gene in each treatment group at different time points, where the expression level at time 0 served as the control, were determined, as shown in Figure 1D–J. Compared with 0 h, *bla_CTX-M-15_* gene expression was upregulated at 2 h in all treatment groups, in which the single-drug groups were upregulated by an average of 5.2, 7.7, and 7.1 times, respectively (Figure 1E,G,I), and the compound groups were upregulated by an average of 4.4, 2.6, and 1.1 times, respectively (Figure 1F,H,J). This indicates that the addition of β-lactam antibiotics potentially increases the relative mRNA expression level of enzyme-producing genes in resistant strains. Excluding the growth control group, the relative expression levels of the *bla_CTX-M-15_* gene in the other groups generally showed a fluctuating pattern over the course of 24 h. As shown in Figure 1F,H,J, the *bla_CTX-M-15_* expression in the CRO/SBT combination group exhibited an increasing trend after 4 h.

**Figure 2:**
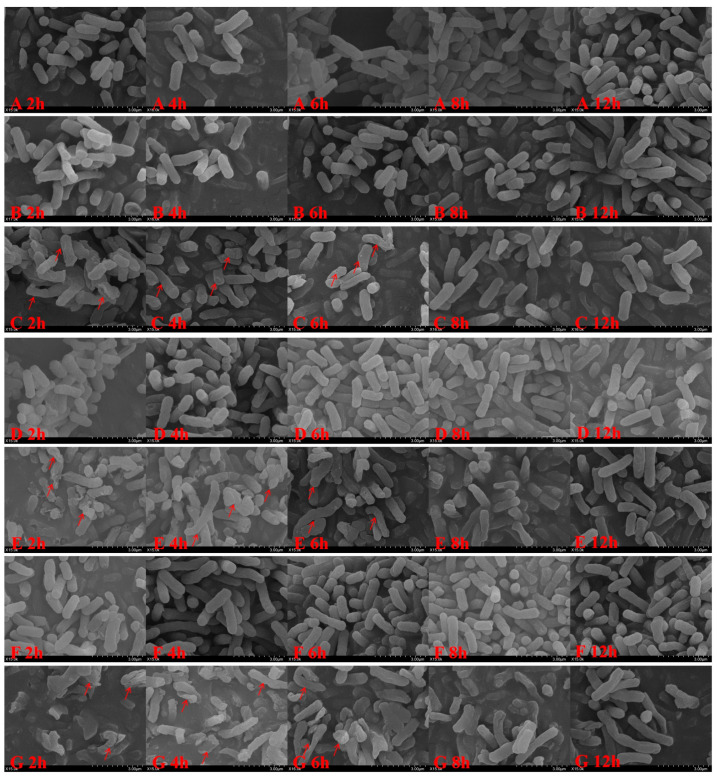
SEM morphology of *Escherichia coli* under different treatments in the postβ-lactamase inhibitor effect experiment. (**A**) Control group; (**B**) CRO 16 µg/mL; (**C**) CRO/SBT 16/8 µg/mL; (**D**) CRO 32 µg/mL; (**E**) CRO/SBT 32/16 µg/mL; (**F**) CRO 64 µg/mL; (**G**) CRO/SBT 64/32 µg/mL.

**Figure 3:**
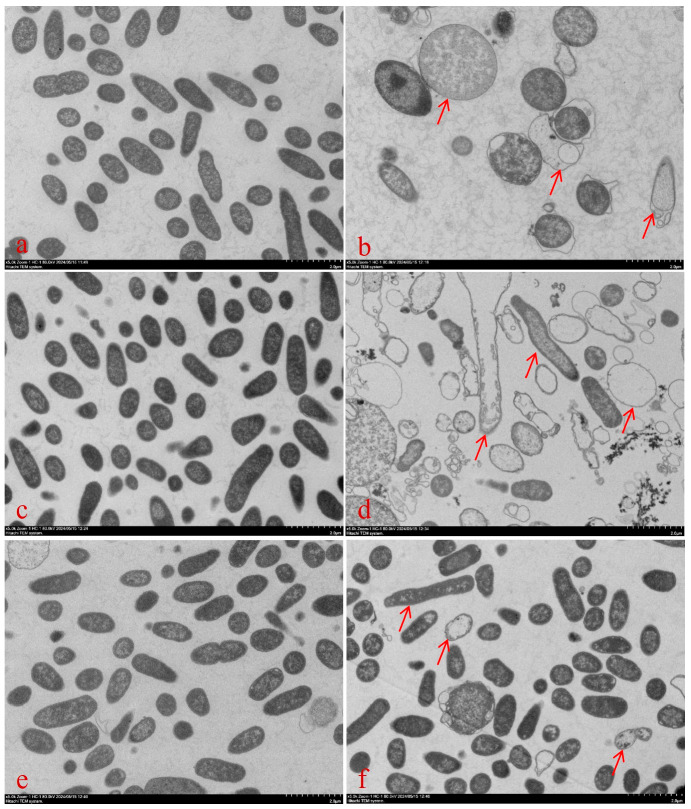
Transmission electron microscopy images showing morphological changes in *E. coli* NCTC 13353 during the postβ-lactamase inhibitor effect (PLIE). (Panels (**a**,**c**,**e**)) correspond to the control group, in which *E. coli* was incubated with 64 μg/mL CRO, with samples collected at 4 h, 8 h, and 12 h, respectively. (Panels (**b**,**d**,**f**)) represent the CRO/SBT combination group (64/4 μg/mL), with samples collected at 4 h, 8 h, and 12 h, respectively.

The microscopy results of the different treatment groups are shown in Figure 2. The scanning electron microscope image shows the surface morphology of *E. coli* at a field of view with a scale of 3 μm. According to the scale ratio, the rod-shaped morphology of individual *E. coli* can be clearly observed. In the control and single CRO treatment groups, *E. coli* maintained a rod-like structure with intact surfaces. In the combination group, at the 2-h time point, most *E. coli* had lost their normal rod-like shape. As shown in Figure 2G, the predominant structures observed were scattered or aggregated bacterial fragments. After SBT removal, some bacteria exhibit uneven thickness, displaying rough surfaces, cracks, or irregular morphological defects, and appear twisted, elongated, shrunken, or swollen under the microscope (as indicated by the red arrows in Figure 2). By 8 h, *E. coli* had recovered to its normal state. These indicate the CRO can perform the antibacterial effect although the SBT remove. The density of some bacteria in the field of view of the transmission electron microscope has significantly decreased, and some bacterial cells have deformed or disintegrated (as indicated by the red arrows in Figure 3). Ultrastructural observations of the CRO/SBT treatment group with a scale of 2 μm revealed that, after SBT removal, CRO alone continued to disrupt the normal structure of *E. coli* and inhibit cell wall synthesis (Figure 3). The PLIE of SBT inhibited β-lactamase activity, thereby enabling CRO to inhibit cell wall synthesis even in the absence of SBT. Overall, SBT can perform PLIE, inhibit β-lactamase activity, and enhancing the antibacterial efficacy of CRO.

### 2.3. Although SBT Is Eliminated from HFIM, SBT Performs PLIE, Which Continuously Inhibits Beta-Lactamase Activity

#### 2.3.1. PK Profile Simulation in the HFIM

The PK parameters for the CRO/SBT combination in plasma were as follows: the t_1/2_, T_max_, k, and C_max_ were 5.878 h, 0.5 h, 0.118 h^−1^, and 29.424 µg/mL for CRO and 1.416 h, 0.5 h, 0.49 h^−1^, and 21.105 µg/mL for SBT, respectively. Compared with the antibacterial effects of CRO administered alone and in combination with SBT, the PK parameters of CRO were the same between CRO alone and the CRO/SBT combinations.

**Figure 4:**
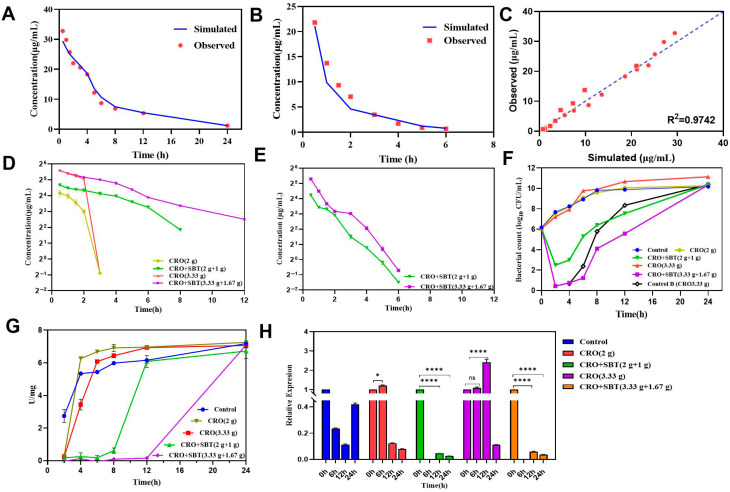
Determination of drug concentrations, bacterial counts, beta-lactamases and gene expression during HFIM under various treatments. (Panels (**A**,**B**)) are the comparisons of HFIM-simulated and observed concentrations for CRO and SBT without bacterial incubation, respectively; (Panel (**C**)) is the linear regression of the simulated and observed concentrations, where R^2^ represents the determination coefficient; (Panels (**D**,**E**)) represent the concentration-time curves for CRO and SBT in HFIM after bacterial incubation; (Panel (**F**)) represents the dynamic time-killing curves under various treatments; (Panel (**G**)) represents the beta-lactamase activity; and (Panel (**H**)) represents the relative *bla_CTX-M-15_* gene expression. * *p* < 0.05, **** *p* < 0.0001 vs. control group.

The drug concentrations simulated by the HFIM followed the same trends as the observed data, which were collected from unpublished phase I clinical trials (Figure 4A,B). Linear regression analysis further demonstrated that the developed HFIM reliably reproduced the PK kinetics of CRO and SBT in plasma under both monotherapy and combination therapy following intravenous (IV) administration (Figure 4C), with a coefficient of determination (R^2^) of 0.974. The drug concentrations of CRO and SBT under IV administration at various doses were measured after bacterial incubation in HFIM, as illustrated in Figure 4D,E. CRO was rapidly eliminated within 3 h during monotherapy, whereas in the CRO/SBT combination therapy, elimination was observed at 8 h in the low-dose group and at 12 h in the high-dose group. The prolonged elimination of CRO in the combination therapy was attributed to SBT inhibiting the degradation of β-lactamase on CRO. Although SBT was eliminated from the HFIM at 6 h, CRO remained in the HF cartridge and was not rapidly degraded by β-lactamase, which indicate the PLIE of SBT.

#### 2.3.2. Dynamic Time-Killing Curves

The dynamic time-killing curves of CRO and CRO/SBT against *E. coli* NCTC 13353 were determined in HFIM, as shown in Figure 4F. The time-kill curves revealed that CRO monotherapy did not have an antibacterial effect. When CRO was combined with SBT at a low dose (2/1 g), the bacterial count was 3.58 log_10_ CFU/mL at 2 h. However, following SBT elimination at 6 h, the bacterial count increased to 5.29 log_10_ CFU/mL, which was comparable to the initial bacterial inoculum. For the high combination dose (CRO/SBT = 3.33/1.67 g), the bacterial concentration was 5.26 log_10_ CFU/mL at 2 h and increased by 4.86 log_10_ CFU/mL at 6 h, indicating a strong correlation between the SBT concentration and the antibacterial effect. During the 0–2 h period, SBT maintained a high concentration in HFIM, and the CRO/SBT ratio remained below 2, allowing the combinations to exhibit synergistic inhibition of *E. coli* growth. However, during the 4–6 h period, as the CRO/SBT ratio exceeded 2, the combinations failed to inhibit bacterial growth. Control group B was subjected to HFIM, where the bacterial count was 1 log_10_ CFU, matching that of the high-dose combination group at the 2-h time point. The growth rates of the control B group and high-dose combination group were determined to be 0.86 and 0.20, respectively, from 4 to 6 h, indicating that SBT continued to inhibit *E. coli* growth even after its elimination.

#### 2.3.3. Determination of β-Lactamase Activity and Expression Levels of the *bla_CTX-M-15_* Gene

For β-lactamase activity (Figure 4G), at the initial stage, the activity in the CRO monotherapy group was lower than that in the control group. This activity gradually increased and eventually surpassed that of the control group, suggesting that β-lactamase activity was initially inhibited due to its degradation of CRO but subsequently resumed its normal activity. In the combination therapy group, β-lactamase activity remained low and gradually increased to a high level, comparable to that in the control group. Figure 4H presents the relative mRNA expression levels of the *bla_CTX-M-15_* gene from HFIM, with the 0-h time point serving as the control. At 6 h, expression increased by 1.21-fold and 1.08-fold in the low-CRO and high-CRO groups, respectively, and by 12 h, it increased by 2.41-fold in the high-CRO group. However, the mRNA expression levels in the CRO/SBT groups were significantly downregulated during the PLIE experiment. Overall, based on growth rate, PK characteristics, and β-lactamase activity, SBT maintains continuous inhibition of β-lactamase activity even after its elimination from HFIM. Moreover, the PLIE increased with increasing SBT concentration.

## 3. Discussion

The CRO/SBT (2:1) combination demonstrates potent antibacterial activity against β-lactamase-producing resistant bacteria in clinical settings. However, the inconsistency in PK has raised concerns regarding its application. In this study, we hypothesized that SBT performs PLIE to inhibit β-lactamase activity and prolong the action of CRO. To comprehensively validate the PLIE, the drug concentration, bacterial count, β-lactamase activity, and *bla_CTX-15_* gene expression were monitored under static conditions and, for the first time, in dynamic HFIM.

CTX-M-type ESBLs are the predominant ESBLs worldwide and are capable of transferring ESBL-carrying plasmids across bacterial species through horizontal gene transfer [25,26]. Among these strains, CTX-M-15 has emerged as the most prevalent ESBL. The MIC of *E. coli* NCTC13353, a bacterium that produces the CTX-M-15 enzyme, to CRO was >1024 μg/mL, whereas the CRO/SBT (2:1) combination could reduce the MIC value to 32 μg/mL, suggesting that SBT could restore the antimicrobial activity of CRO, which was in line with the results of other studies on the antimicrobial activity of CRO/SBT [14,17,27]. However, the inconsistencies in PK properties, particularly the differing half-lives of CRO (5.88 h) and SBT (1.42 h) in plasma, pose challenges for clinical application.

PLIE involves several β-lactamase inhibitors that continue to inhibit enzyme activity after they are eliminated [21]. Lavigne et al. determined the PLIE of ceftazidime-sulbactam against *K. pneumoniae* and *E. coli* strains expressing different ESBL variants, reporting durations ranging from 0.7 to 5 h [23]. Sader et al. explored the PLIE of ceftolozane-tazobactam against *E. coli* producing CTX-M-15 ranging from 1.3–2.1 h [22]. In the static experiment of the present study (Figure 1), the PLIE was found for CRO/SBT combinations, as bacterial regrowth occurred after 2 h, while CRO concentrations remained stable and β-lactamase activity did not increase from 2–4 h after SBT removal in CRO/SBT combination treatments, indicating sustained inhibition of the enzyme. In the 2 MIC group (CRO/SBT: 64/32 μg/mL), PLIE was positive (0.37 h), while PLIE values were −1.26 h in the 1/2 MIC (CRO/SBT: 16/8 μg/mL) and −0.57 in the 1 MIC groups (CRO/SBT: 32/16 μg/mL). Pillar et al. also reported a negative PLIE at low concentrations, where a low-concentration drug binds to PBP3, catalyzing septum formation during bacterial division and primarily inhibiting division under β-lactam exposure [24]. This results in the formation of filaments instead of typical division [28,29]. Upon drug removal, the filaments separate, leading to rapid bacterial proliferation and a negative PLIE phenomenon. In addition, the dependence of the PLIE values on drug concentrations was determined, which is consistent with previous findings.

HFIM is a useful tool for investigating the dose-response relationship considering PK profiles. Only free drugs have antimicrobial activity [30]; in this study, plasma protein binding rates of 89.5% and 38% for CRO and SBT, respectively, were adapted for the development of HFIM [31,32]. In the current study, as Figure 4C shows, the HIFM-simulated data can capture the PK profiles of CRO and SBT after the combination formulation treatment. The dose-response relationships of CRO monotherapy and CRO/SBT (2:1) combination therapy against *E. coli* NCTC13353, as well as the PLIE of the combinations, were determined by HFIM in this study. When NCTC13353 was exposed to the high-dose combination group of CRO/SBT (3.33/1.67 g), the maximum reduction was approximately 87% of the initial inoculum at 2 h, and a significant decrease in β-lactamase activity compared with that of the control was observed. When *E. coli* NCTC13353 was exposed to the low-dose combination group (2/1 g), the bacterial percentage was approximately 40% of the initial inoculum at 2 h, and β-lactamase activity was significantly decreased. As SBT was eliminated at 6 h, CRO was still maintained at a certain concentration for 6–12 h in the low-dose group and for 6–24 h in the high-dose group, which is different from the monotherapy group, where CRO was rapidly eliminated. In addition, β-lactamase activity remained low at 6–8 h (low-dose combination group) and 6–24 h (high-dose combination group). *E. coli* NCTC13353 resumed growth and produced β-lactamase, leading to a gradual decline in CRO concentration. Eventually, when the CRO concentration decreased to zero, the β-lactamase became “free” and displayed high activity at 12 and 24 h. In addition, the results of fluorescent quantitative PCR of bacterial β-lactamase genes revealed that even at the end of the 24-h trial, the expression level of bacterial enzyme-producing genes in the CRO/SBT group was lower than that in the CRO group.

For the high-dose combination group, CRO/SBT consistently maintained the bacterial density below 1.2 log_10_ CFU/mL from 2–6 h. For the low-dose group, also referred to as the label dose, the maximum simulated concentration of CRO/SBT was below the MIC and unable to inhibit bacterial growth, indicating that the drug dosage needs to be optimized. HFIM can be further utilized to explore optimal dosing regimens for CRO/SBT, including dosage amounts, ratios, and intervals. Tam et al. applied the HFIM to evaluate the PK/PD relationship and optimize the dosing strategies of various beta-lactam/beta-lactamase inhibitor combinations, such as piperacillin/tazobactam and ceftazidime/avibactam against ESBL-producing bacteria under high-inoculum conditions [33]. Moreover, HFIM can be used to investigate the emergence of resistance mechanisms and the antibacterial effects on biofilm formation.

The main factors influencing PLIE include bacterial characteristics (e.g., growth rate, susceptibility, and enzyme production rate), β-lactamase types, and treatment strategies (including drug combinations and duration of therapy). For example, *E. coli* and *Klebsiella pneumoniae*, which produce the same type of ESBL, exhibit similar PLIEs when treated with the same drugs [23]. Pillar et al. reported that the same β-lactamase inhibitor, when combined with different β-lactam antibiotics, resulted in varying PLIE values for the same bacterial species [24]. Nakae et al. reported that in Pseudomonas aeruginosa, efflux pump overexpression occurs when β-lactamase is inhibited [34]. In our study, SBT inhibited β-lactamase, possibly inducing efflux pump overexpression that expels CRO, reducing its accumulation and antibacterial effect after SBT removal. Further research is needed to clarify the relationship between efflux pumps and PLIE.

Based on changes in bacterial count, β-lactamase activity, and relative gene expression, we found that the SBT concentration—based PLIE can preserve the protective effect for CRO, preventing it from being degraded by β-lactamase. However, this study has several limitations. Our research primarily focused on the phenotypic level, with limited investigation into the underlying mechanisms. In future work, the *bla_CTX-M-15_* gene should be cloned for recombinant expression to clarify the contribution of β-lactamase through in vitro β-lactamase inhibition kinetics and metabolomics analysis. Moreover, the role of efflux pumps requires further investigation, and additional wild *E. coli* isolates producing CTX-M-15 β-lactamase should be tested to assess potential genetic variants. Finally, as all PLIE assessments in this study were performed in vitro, the in vivo characteristics of PLIE for SBT and the influence of host immune factors on bacterial inhibition remain unclear. Future studies should develop in vivo murine infection models to better characterize PLIE under physiological conditions.

In conclusion, the CRO/SBT combination has a significant postβ-lactamase-inhibitor effect on enzyme-producing strains by maintaining β-lactamase inhibition and increasing the activity of CRO.

## 4. Materials and Methods

### 4.1. Antimicrobial Agents and Bacteria

Ceftriaxone sodium (purity of 91.5%) and sulbactam sodium (purity of 91.6%) were obtained from Xiangbei Welman Pharmaceutical Co., Ltd. (Liuyang, China). Nitrocephin (purity of 99%) was obtained from Shanghai Aladdin Bio-Chem Technology Co., Ltd. (Shanghai, China).

*E. coli* NCTC 13353, which produces CTX-M-15 ultrabroad-spectrum β-lactamase, was purchased from the United Kingdom Health Security Agency; *E. coli* ATCC 25922 was provided by the National Reference Laboratory of Veterinary Drug Residues at Huazhong Agriculture University [35].

### 4.2. Antimicrobial Susceptibility Testing

Following CLSI guidelines [35], the minimum inhibitory concentrations (MICs) of various drug treatments, including CRO alone, the combination of CRO/SBT at a ratio of 2:1, and the combination of CRO/SBT with a fixed SBT concentration of 4 μg/mL, against *E. coli* NCTC 13353 were determined via the broth microdilution method. Serial twofold dilutions of CRO, ranging from 16 to 2048 μg/mL, were prepared and mixed with *E. coli* NCTC 13353 (at a concentration of 10^6^ CFU/mL) in 96-well plates. The 96-well plates were incubated at 37 °C for 16 to 20 h to determine the MIC. The MIC of CRO against *E. coli* ATCC 25922 was used as the quality control standard.

### 4.3. Static Postβ-Lactamase Inhibitor Effect (PLIE)

The postβ-lactamase inhibitor effect (PLIE) was determined on the basis of a previous study [21]. Briefly, *E. coli* NCTC 13353 (1 × 10^6^ CFU/mL) was exposed to various drug treatments, including the control, CRO single groups at concentrations of 1/2, 1, and 2 × MIC, and CRO/SBT combination groups (at a 2:1 ratio) with CRO concentrations of 1/2, 1, and 2 × MIC, respectively, at 37 °C for 2 h. After incubation, the drug and supernatant were removed by centrifugation at 3000 r/min for 15 min, followed by washing twice with an equal volume of fresh LB medium. Subsequently, 50 mL of LB medium containing only the corresponding CRO concentrations was added, and the cultures were incubated at 37 °C. Samples were collected at 0, 2, 4, 6, 8, 12, and 24 h for bacterial count (0.3 mL), drug concentration determination (0.2 mL), and morphological assessment (5 mL). The viable bacterial counts were determined after 24 h of incubation. PLIE was calculated via Equation (1).

(1) $$PILE=\left(t_{combination}-t_{control}\right)-\left(t_{single}-t_{control}\right)$$

where *t* represents the time required for a 1 log_10_ CFU/mL increase in the bacterial count. *t_combination_* and *t_single_* refer to the times observed in the combined and single treatment groups at the same ceftriaxone concentration, respectively, whereas *t_control_* represents the time observed in the control group.

#### 4.3.1. Determination of Bacterial Count

The samples were serially diluted 10-fold in drug-free medium, and 100 μL of the appropriate dilution was spread on LB agar plates placed in an incubator at 37 °C for 20 h to determine the number of viable bacteria (n = 3).

#### 4.3.2. Determination of Ceftriaxone and Sulbactam Concentrations

High-performance liquid chromatography-tandem mass spectrometry (HPLC-MS/MS) with an HPLC system (LC-20AD, SHIMADZU, Kyoto, Japan) connected to a triple quadrupole mass spectrometry detector (AB Sciex API 5000, Applied Biosystems, Foster City, CA, USA) was used to determine the ceftriaxone and sulbactam concentrations in the LB broth. For sample preparation, 25 μL of the sample was acidified by adding 625 μL of methanol and 600 μL of 0.1% formic acid in water. The mixture was vortexed for 1 min and centrifuged at 8000 r/min for 10 min. The filtered supernatants were eluted at a flow rate of 0.3 mL/min through a Hypersil GOLD C18 analytical column (150 × 2.1 mm, 5 µm; Thermo Fisher Scientific, Waltham, MA, USA) via a mobile phase gradient consisting of mobile phase A (0.1% formic acid in water) and mobile phase B (0.1% formic acid in acetonitrile). Gradient elution was applied as follows: 0–1 min, 5% mobile phase B; 3–5 min, 95% mobile phase B; and 5.1–8 min, 5% mobile phase B. The injection volume was 5 μL.

The electrospray ionization mode used was the renin negative mode (ESI-), and the MRM mass spectrometric parameters of ceftriaxone and sulbactam are listed in Table 1. The detected ranges of both ceftriaxone and sulbactam were between 5 and 500 μg/L, with a strong linear correlation (r > 0.99). The recoveries of ceftriaxone and sulbactam in LB broth medium under this method ranged from 86.8% to 101.3% and 95.8% to 109.0%, with intrabatch coefficients of variation ranging from 1.4% to 7.8% and 0.9% to 7.2%, respectively, and interbatch coefficients of variation ranging from 3.0% to 8.7% and 5.6% to 6.1%, respectively.

#### 4.3.3. Determination of β-Lactamase Activity

β-Lactamase activity was determined during PLIE via the nitrocefin test. The samples (1 mL) collected from HFIM were centrifuged at 8000 r/min for 10 min and resuspended in 1 mL of precooled PBS buffer (4 °C). The samples were then ultrasonically disrupted at 35% power for 10 s, followed by a 15-s interval, which was repeated for 3 min while the samples were kept on ice. The mixture was centrifuged at 4000 r/min for 5 min, and the supernatant (containing the crude enzyme mixture) was collected for protein quantification via a BCA kit (Nanjing Vazyme Biotech Co., Ltd., Nanjing, China). To measure enzyme activity, 0.26 mL of PBS buffer, 0.03 mL of 10^−4^ mol/L nitrocefin, and 0.01 mL of enzyme mixture were added to a 96-well plate and incubated at 37 °C for 10 min. Enzyme activity was determined on the basis of the change in absorbance at 482 nm.

#### 4.3.4. RT-qPCR for Quantification of *bla_CTX-M-15_*

Modified TRIzol methods were used to extract high-quality genomic RNA from bacterial samples. Genomic DNA was removed from the RNA samples via a reverse transcription kit (Vazyme Biotech Co., Ltd., Nanjing, China), after which reverse transcription was performed to obtain cDNA for qPCR. RT-qPCR was conducted using a Bio-Rad IQ5 instrument (Bio-Rad, Hercules, CA, USA). Each reaction mixture included cDNA (1.5 μL), forward and reverse primers (0.8 μL), SYBR qPCR Master Mix (10 μL) and ddH_2_O (7.7 μL). The cycling conditions were as follows: an initial denaturation step at 95 °C for 30 s, followed by 44 cycles at 95 °C for 10 s and 57 °C for 30 s. The melting curves were generated from 65 °C to 95 °C at 0.5 °C increments with a dwell time of 5 s at each temperature. The expression of *bla_CTX-M-15_* was quantified relative to the internal reference gene 338f/806r by normalizing the cycling threshold (Ct) values. The primer sequences were as follows: q338 forward primer, 5′-ATGTGCAGYACCAGTAARGTKATGGC-3′; q806 reverse primer, 5′-TGGGTRAARTARGTSACCAGAAYSAGCGG-3′; *bla_CTX-M-15_* forward primer, 5′-AGCGATAACGTGGCGATGAATAAG-3′; and reverse primer, 5′-CGGTACGGTCGAGACGGAAC-3′.

#### 4.3.5. Bacterial Morphology

Morphological changes in *E. coli* NCTC 13353 during PLIE, following coincubation with CRO or CRO/SBT, were analyzed by scanning electron microscopy (SEM) (JSM-6390SV, Komatsu NTC Ltd., Nanto, Japan) and transmission electron microscopy (TEM) (HT7800, HITACHI, Tokyo, Japan). The samples were centrifuged to remove the supernatant and then fixed with 2.5% glutaraldehyde for 3 h. Dehydration was performed using gradient ethanol (30%, 50%, and 70%) for 20 min each. Following vacuum freeze-drying for 24 h, the samples were sputter-coated with the Au membrane for SEM imaging.

For TEM imaging, 8 mL of *E. coli* NCTC 13353 was added to 72 mL of drug-containing medium to achieve final concentrations of 64 μg/mL for the CRO group and 64/4 μg/mL for the CRO/SBT combination group. The mixtures were incubated at 37 °C for 4 h. After the initial 4-h incubation, all the drug-containing media were removed via centrifugation and washing. The bacteria were then resuspended in an equal volume of fresh medium containing CRO (64 μg/mL), and incubation was continued at 37 °C for an additional 8 h. Thus, the total incubation time was 12 h. The samples were collected at three time points: 4 h (before drug removal) and 8 and 12 h (after drug replacement). The collected samples were fixed in 2.5% glutaraldehyde for 24 h, followed by fixation with 1% osmium acid. After three washes with 0.1 M phosphate buffer, the samples were dehydrated via a graded acetone series (30%, 50%, 70%, 80%, and 95%) and subjected to osmotic embedding with a mixture of acetone and embedding agent at 37 °C. Polymerization was carried out at 60 °C for 48 h. The embedded resin blocks were then ultrathinly sectioned, stained, and prepared for TEM observation.

### 4.4. Hollow-Fiber Infection Model (HFIM) Simulating the In Vivo PK of CRO Monotherapy and CRO/SBT Combinations

#### 4.4.1. Development of the HFIM

**Figure 5:**
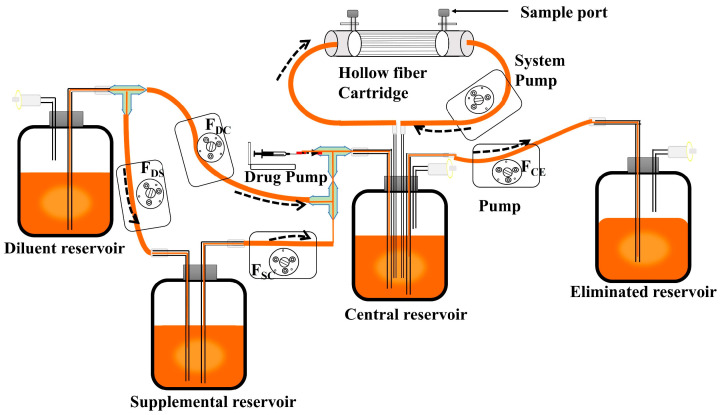
Schematic diagram of the hollow fiber infection model (HFIM) for combination therapy. HFIM consists of a diluent, a supplemental, central, or eliminated reservoir and an HF cartridge. Bacteria were incubated in the HF cartridge via the sample port and retained outside the fiber, while the drug and nutrients were equilibrated between the central reservoir and the HF cartridge. Fresh medium was continuously supplied to the central reservoir at a fixed rate from the diluted reservoir, while the contents were simultaneously removed to the elimination reservoir at the same rate via a pump.

HFIM was used to evaluate changes in bacterial burden, β-lactamase activity, and morphology under dynamic drug concentrations following various dosage regimens: (i) ceftriaxone alone at 2 g; (ii) low-dose COR/SBT at 2 g/1 g; (iii) COR alone at 3.33 g; and (iv) high-dose COR/SBT at 3.33 g/1.67 g. The administration route is intravenous infusion for a single dose. A schematic structure of the HFIM used in this study is shown in Figure 5.

HFIMs for CRO alone and the CRO/SBT combination are respectively developed. The HFIM for CRO alone consists of a diluent reservoir, an elimination reservoir, a central compartment, a hollow fiber Cartridge (FB-170 polysulfone cartridge, Hubei Science Laboratory Equipment Co., Ltd., China), also termed the peripheral compartment, peristaltic pumps (Baoding qili precision pump Co. Ltd., China), and a magnetic stirrer (SCI550-Pro, Scilogex, Rocky Hill, CT, USA). The HFIMs for the single drugs used in the current study have been described in previous studies [36]. Briefly, *E. coli* NCTC 13353 at a concentration of 1 × 10^6^ CFU/mL was maintained in the peripheral compartment, from which bacteria cannot enter the system. At the beginning of the experiment, the system pump was activated. CRO was injected at a rate of 0.5 mg/min for 30 min via an injection pump (LSP01-1Y, Baoding Rongbo Constant Flow Pump Manufacturing Co., Ltd., China). Fresh LB medium and drugs can circulate through a central compartment into and out of the peripheral compartment under system pump drive. After 30 min of infusion, the injection pump was turned off, and all peristaltic pumps were activated. The pump frequency from the diluent reservoir to the central reservoir (F_DC_) and from the central reservoir to the eliminated reservoir (F_CE_) is equivalent to simulating CRO elimination. The samples were collected from the sampling points of the HF cartridge for pharmacokinetic and pharmacodynamic analysis.

As shown in Figure 5, in addition to the components used for the single-drug HFIM, one supplemental reservoir and two additional pumps were incorporated into the HFIM for the CRO/SBT combination. Total clearance was represented by the peristaltic pump frequency from the central reservoir to the elimination reservoir (F_CE_), which was determined on the basis of the elimination rate constant of the SBT. The pump frequency from the diluent reservoir to the central reservoir (F_DC_) was set according to the elimination rate constant of CRO. Since SBT has a shorter half-life and a higher elimination rate constant than CRO does, F_CE_ was significantly greater than F_DC_. To simulate the PK profile of CRO, a compensatory solution containing CRO (A_S,CRO_) was transferred from the supplemental reservoir to the central reservoir at a pump frequency (F_SC_), which was calculated as F_CE_ − F_DC_. Furthermore, to maintain the balance for supplemental reservoir, the pump frequency from the diluent reservoir to the supplemental reservoir (F_DS_) was set equal to the F_SC_. This model effectively preserved overall fluid balance and simultaneously simulated the pharmacokinetic profiles of two antibiotics with different half-lives. At 0 min, the injection pump and system pump were turned on, and CRO and SBT were infused into the central reservoir for 30 min, during which only the three-way valve connecting the injection pump to the central reservoir was open. After 30 min of infusion, the injected pump was turned off the other pumps were activated, and the three-way valve was switched to connect the diluent reservoir, supplementary reservoir, and central reservoir.

#### 4.4.2. Simulated PK Profiles in HFIM

The time–concentration data of CRO and the CRO/SBT combination were obtained from an unpublished phase I clinical trial conducted by the National Drug Clinical Research Base of Xijing Hospital, Fourth Military Medical University. The Clinical Trial approval number is 2005L00728. The subjects are 30 healthy volunteers, half male and half female, aged 18–50 years with normal heart, lung, liver, and kidney function, no history of Penicillin allergy, and a body mass index between 19–24 kg/m^2^ who voluntarily participate in this Clinical Trial. The reproduction of PK data in HFIM by adjusting drug amount, system volume and pump rate based on the PK parameters. Pharmacokinetic (PK) parameters, including the half-life (t1/2), time of maximum concentration (Tmax), elimination rate constant (k), and maximum concentration (Cmax), were calculated via a noncompartmental model implemented in Phoenix (version 8.3, Certara, Radnor, PA, USA). The protein binding rates of CRO and SBT in plasma were 0.895 and 0.38, respectively, which were used in HFIM development to simulate free drug concentrations in plasma. The PK parameters are input into the web dashboard [37] to determine the relevant parameters for HFIM development, including the volumes of the reservoirs, pump rates, and amount of drug. The HFIM setting parameters, including pump rates, reservoir volumes, and added drug amounts, for CRO monotherapy and CRO/SBT combination therapy are provided in Table 2.

#### 4.4.3. Determination of Bacterial Number, β-Lactamase Activity, and Expression Level of *bla_CTX-M-15_*

Samples were collected from the sampling port of the HF cartridge at various time points after drug administration. At 0, 2, 6, 8, 12, and 24 h, 6 mL of sample was collected, of which 5 mL was used for RNA extraction and *bla_CTX-M-15_* gene expression. The remaining 1 mL was used for simultaneous determination of the viable bacterial count, β-lactamase activity, and drug concentration. For PK analysis, additional 1 mL samples were collected at 0.5, 1, 1.5, 3, 4, and 5 h. All drug concentrations were measured via HPLC-MS/MS. The viable bacteria count, drug concentration, β-lactamase activity, and expression level of *bla_CTX-M-15_* were determined as described above.

### 4.5. Statistical Analysis

Each measurement, including drug concentration, bacterial count, CTX-M-15 gene expression, and β-lactamase activity, was performed in triplicate in both the static PLIE and HFIM experiments. Differences between groups were assessed using two-factor ANOVA followed by Tukey’s post hoc test, with statistical significance set at *p* < 0.05. All the statistical analyses were conducted via GraphPad Prism 8.0.

## Figures and Tables

**Table 1:** MRM mass spectrometric parameters of ceftriaxone and sulbactam.

Drug	Quantitative Ion Pair (*m*/*z*)	Qualitative Ion Pair (*m*/*z*)	Declustering Potential (DP/V)	Collision Energy (U/V)
Ceftriaxone	555.1 > 396.0	555.1 > 396.0	110	30
		555.1 > 324.0		
Sulbactam	232.1 > 64.0	232.1 > 64.0		18
		232.1 > 140.1		

**Table 2:** Parameter settings for the HFIM of ceftriaxone and ceftriaxone/sulbactam.

Group	Parameters	Calculation Equation	Value
CRO-SBT	V	-	500.0 mL
	F_CE_	F_CE_ = V × k_SBT_	4.1 r/min
	F_DC_	F_DC_ = V × k_CRO_	1.0 r/min
	F_DS_/F_SC_	F_DS_ = F_CE_ − F_DC_	3.1 r/min
	V_S_	V_S_ = F_M/N_/k_CRO_	1577.6 mL
	A_CRO_	A_CRO_ = C_max,CRO_ × V	15.0 mg
	A_SBT_	A_SBT_ = C_max,SBT_ × V	10.6 mg
	A_S,CRO_	A_S,CRO_ = A_CRO_ × (k_SBT_/k_CRO_ − 1)	46.4 mg
	R_CRO_	R_CRO_ = A_CRO_/T	0.5 mg/min
	R_SBT_	R_SBT_ = A_CRO_/T	0.35 mg/min
CRO	V	-	500.0 mL
	F_DC_	FDC = V × k_CRO_	1.0 r/min
	F_CE_	FCE = V × k_CRO_	1.0 r/min
	A_CRO_	A_CRO_ = C_max,CRO_ × V	15.0 mg
	Rate_CRO_	R_CRO_ = A_CRO_/T	0.5 mg/min

A_CRO_ represents the amount of ceftriaxone injected, whereas A_SBT_ represents the amount of sulbactam injected. A_S,CRO_ denotes the initial amount of ceftriaxone in the supplementary reservoir. C_max,CRO_ and C_max,SBT_ refer to the maximum plasma concentrations of ceftriaxone and sulbactam, respectively. F_CE_ represents the peristaltic pump frequency from the central reservoir to the eliminated reservoir, whereas F_DC_ represents the peristaltic pump frequency from the diluted reservoir to the central reservoir. F_DS_ represents the peristaltic pump frequency from the diluted reservoir to the supplementary reservoir, and F_SC_ represents the peristaltic pump frequency from the supplementary reservoir to the central reservoir. k_CRO_ and k_SBT_ represent the elimination rates of ceftriaxone and sulbactam, respectively. R_CRO_ and R_SBT_ represent the injection rates of ceftriaxone and sulbactam, respectively. T represents the injection period, which is 30 min. V represents the volume of the central reservoir and HF cartridge, whereas V_S_ represents the volume of the supplementary reservoir.

## Data Availability

Data are contained within the article.
